# Different cardiorespiratory fitness expressions based on the maximal cycle ergometer test show no effect on the relation of cardiorespiratory fitness to the academic achievement of nine-year-olds

**DOI:** 10.1371/journal.pone.0200643

**Published:** 2018-07-23

**Authors:** Elvar Saevarsson, Erla Svansdottir, Sigurbjorn Arngrimsson, Thorarinn Sveinsson, Erlingur Johannsson

**Affiliations:** 1 School of Education, University of Iceland, Reykjavík, Iceland; 2 Research Centre for Movements Sciences, University of Iceland, Reykjavík, Iceland; 3 Department of Sport and Physical Activity, Western Norway University of Applied Sciences, Bergen, Norway; Universita degli Studi di Verona, ITALY

## Abstract

The relationship between cardiorespiratory fitness and academic achievement has been inconclusive. The results may depend on how cardiorespiratory fitness is expressed. The aim of this study is to explore the impact of different cardiorespiratory fitness expression methods, measured by the maximal cycle ergometer test, on the relationship between cardiorespiratory fitness and academic achievement. A cross-sectional study consisting of 303 Icelandic 4^th^ grade students (163 girls) was conducted. Cardiorespiratory fitness was assessed using a graded maximal cycle ergometer test and scores of standardized tests in Icelandic and math obtained from the Icelandic National Examination Institute. Cardiorespiratory fitness was measured as absolute power output in watts in a maximal progressive cycle ergometer test. To adjust for different body sizes, the power output was scaled to body weight, body height, body surface area, and allometrically expressed body weight. In addition, linear regression scaling was also used to adjust for different body sizes. No significant relationship was found between any of the cardiorespiratory fitness expressions and academic achievement, using both univariate and multivariate linear regression analyses. The use of different methods to express cardiorespiratory fitness does not significantly affect the association with the academic achievement of fourth grade students.

## Introduction

Academic achievement is an important influential factor in the future educational attainment and health of individuals and has therefore been viewed as a public health concern [1]. Compared to the poorly educated, the well-educated are less likely to smoke and are more likely to exercise, to get health check-ups and to drink moderately, all of which are associated with good health [2]. Given the importance of academic achievement and the resulting educational attainment for future health and well-being, it is imperative to understand the determinants of academic achievement.

In addition to background family factors, including socioeconomic status, lifestyle-related factors, such as physical activity and obesity, have been linked to academic achievement [3,4]. Cardiorespiratory fitness (CRF) is defined as the ability to carry out daily tasks with vigour and alertness without undue fatigue and with ample energy to enjoy leisure-time pursuits and respond to emergencies [5]. CRF has been considered to be a proxy for the level of intensive physical activity of recent months among youths [6]. The relation between CRF and academic achievement has been studied to some extent, but the results thus far are inconclusive. In a study involving 38992 third to 12^th^ graders, Welk et al. reported CRF to be associated with achievement in math and reading [7], and Rauner et al. [8] found CRF to predict the outcomes in math and reading in 10 to 14-year-old students. Contrary to the aforementioned studies, Haapala et al. [9] did not find CRF to be associated with academic achievement in reading and math of six- to eight-year olds, and Coe et al. [10] reported no relations between CRF and achievement in math, English, and social studies among sixth and ninth graders.

One possible explanation for the discrepancies is the method used to express CRF. Since a larger and heavier body can produce more absolute power, the importance of adjusting the absolute power values for different body sizes is well recognized. This has most commonly been done using a ratio scaling by expressing CRF relative to body size (W/kg). The literature indicates that adjusting for different body sizes impacts the association between CRF and academic achievement. Kwak et al. reported positive findings with CRF test results expressed as an absolute power output (W) [11]; on the contrary, Kantomaa et al. did not find any relation between CRF and academic achievement when CRF test results were expressed relative to body size (peak oxygen uptake in ml/kg/min predicted based on heart rate reactions during a submaximal cycle ergometer test) [12]. This may indicate that either body size confounds the association between CRF and academic achievement or the use of ratio scaling leads to spurious results [13].

Previous studies have described the importance of proper scaling of CRF in relation to cardiometabolic risk factors in children [14,15], but the relation of different CRF scaling to academic achievement has not been studied. The aim of this study was therefore to explore whether the way CRF is expressed affects the relationship between CRF, measured by a maximal cycle ergometer test, and academic achievement.

## Materials and methods

**Fig 1:**
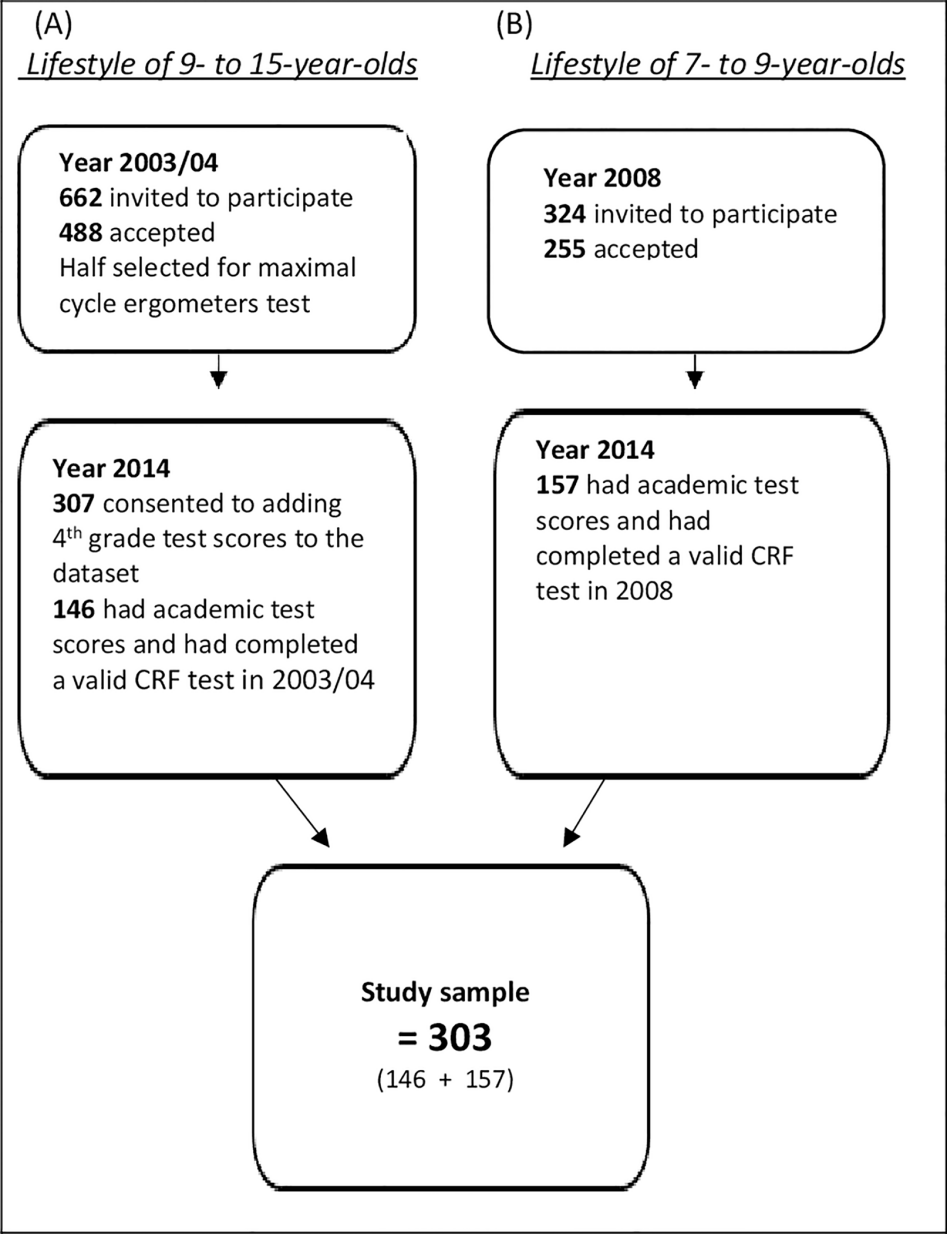
The origins of the study sample. Participants came from two different study cohorts, “Lifestyle of 9- to 15-year-olds” and “Lifestyle of 7- to 9-year-olds.” The participation rate, inclusion criteria and total number of participants are displayed.

The study population stems from two different epidemiological studies conducted in Iceland involving nine-year-old children. The first study, *“Lifestyle of 9- and 15-year-old Icelandic children”* [16], included nationally representative data collected from children born in 1994 (n = 662 invited to participate, n = 488 accepted) between September 2003 and January 2004. It was part of the European Youth Heart Study (EYHS) [17]. Half of the cohort were randomly selected and invited to participate in the CRF test. The Ethics Committee waived the requirement for consent to add fourth (9-year-old) grade test results to the database, and so the participants were contacted in 2014, at age 20, by mail and phone and were asked for their written informed consent to add their academic achievement to the database. Of those who consented to participate (n = 307), 146 possessed academic test scores and had completed a valid CRF test in 2003/04 (Fig 1).

The second study, *“Lifestyle of 7–9-year-olds*: *Intervention toward better health”* [18], was conducted in 2006–2008. It involved children born in 1999 (n = 324 invited to participate, n = 255 accepted) from the nation’s capital, Reykjavik. Written informed consent from legal guardians to add academic achievement from 2008 to the database was obtained in 2008. The last phase of the data collection (used in this study) was conducted from late August to early December 2008. The total number of participants in both studies with the information required for statistical analysis in the current study was n = 303 (Fig 1).

The National Bioethics Committee in Iceland approved the study (VSNa2003060014/03-12/BH/–).

### Cardiorespiratory fitness

CRF was estimated from performance results on a graded maximal exercise test on an electronically braked cycle ergometer (Monark Ergomedic 839, Vansbro, Sweden), according to the EYHS test protocol [17]. Initial and incremental workloads were 20 W for children weighing less than 30 kg and 25 W for those weighing 30 kg or more. The workload was programmed to increase after every 3 min. Heart rate was recorded continuously (Polar Vantage, Kempele, Finland) throughout the test, and the test continued until the subject could no longer continue. The criterion for a valid maximal test was a) heart rate ≥ 185 or b) subjective observation from the researcher that the child could not continue (after vocal encouragement if necessary). If the pedalling rate dropped below 30 rpm, the child was deemed to have stopped the test. The absolute power output from this cycle ergometer test correlates very well with measured maximal oxygen consumption (VO2 max) in 9- to 11-year-old children, r = 0.90 and r = 0.95, for boys and girls, respectively [19].

### Anthropometrics

Height (cm) and weight (kg) were measured to the nearest 0.1, using standard procedures allowing for the calculation of body mass index (BMI—kg/m^2^). Body surface area (m^2^) was determined using the equation of Haycock et al. [20]. Maturity level was estimated using the newly developed sex specific equations which estimate years from/to peak height velocity, i.e., maturity offset (-7.99994 + (0.0036124 * age * height) for boys and -7.709133 + (0.0042232 * age * height) for girls) [21].

### Academic achievement

Grades from standardized tests in Icelandic and 4^th^ grade math were obtained from The National Examination Institute and used to assess academic achievement. The National Examination Institute develops and administers the tests once a year in September to all students attending fourth, seventh and tenth grades in the country to assess their standing in Icelandic and math. The test in Icelandic estimates performance in reading, writing, and grammar and comprises multiple choice questions, reading comprehension, short story writing, and the correct use of words. The test in mathematics comprises multiple choice questions, word problems, sentence completion, operations, geometry, and numerical understanding [22,23]. The nation-wide results were normalized with a mean of 30, standard deviation of 10, and total score range of 0–60 [22,23]. The average score from the two academic subjects was used in this study. Socioeconomic status was estimated with a dichotomous variable, with 1 representing one or both parents having a university degree.

### Statistical analysis

The data was analysed using SPSS 22.0 software (IBM Corp., Armonk, NY, USA) and reported as the mean ± SD. All variables were inspected for normality. Body weight and CRF, expressed relative to body weight, were log-transformed to correct for skewness (logW/kg and logkg) [24]. CRF was expressed as absolute power output (Max W) and power output relative to body size (W/kg), height (W/cm), body surface area (W/BSA), body weight expressed allometrically to the exponent 0.66 (W/kg^0.66^) [25], and body weight expressed allometrically to the calculated sample’s exponent (0.51 and 0.35 for girls and boys, respectively). Allometric modelling has been reported to be useful for exercise data as it incorporates a multiplicative, rather than additive, error term and thus controls for heteroscedasticity [26]. The numeric value of the parameters was solved by applying a least square regression to the linear form of the allometric model, log_e_ Y = log_e_ a + b * log_e_ ɛ [26]. For these normalizations to be considered valid, the intercept from the regression of the adjusting variable to the absolute power output must not be significantly different from zero [13]. To explore the validity of each normalization, linear regression models were performed for each adjusting variable. To eliminate the issue of a potential non-zero intercept, a separate linear regression model was performed with power output and body weight as the predictors and academic achievement as the response variable (linear regression scaling) [26]. One-way ANOVA (continuous variables) and chi-square (categorical variables) tests were used to explore the mean differences on key variables between the sexes and between the study sample and the excluded participants. Univariate and multi-variate linear regressions were used to explore the relation between different CRF expressions and academic achievement. All analyses were stratified by sex because strong relationships between CRF and sex and between academic achievement and sex have previously been reported [22,27]. Statistical significance was accepted at an α level < 0.05.

## Results

Table 1 presents the descriptive characteristics of the study sample and the rest of the cohort. The included and excluded participants differed on all CRF expressions. This was expected since a valid maximal CRF test was part of the inclusion criteria. The study sample was also significantly taller than the rest of the sample. The boys had significantly higher CRF levels than the girls on all CRF expressions, but the girls had significantly higher test scores and were more mature (closer to the age of peak height velocity) (see Table 1).

**Table 1:** Physical characteristics of the study sample and excluded participants.

Characteristic	Boys (n 140) mean ± SD	Girls (n 163) mean ± SD	Sample (n 303) mean ± SD	Excluded (n) mean ± SD
BMI	17.49 ± 2.58	17.38 ± 2.42	17.43 ± 2.50	17.50 ± 2.84 (259)
Weight (kg)	34.02 ± 6.56	33.49 ± 6.30	33.73 ± 6.41	33.16 ± 7.40 (259)
Height (cm)	139.11 ± 5.27	138.40 ± 5.93	138.73 ± 5.64	137.07 ± 6.40* (259)
BSA (m^2^)	1.14 ± 0.13	1.13 ± 0.13	1.13 ± 0.13	1.12 ± 0.15 (259)
Max W	107.94 ± 18.81	91.05 ± 19.15**	98.86 ± 20.75	81.19 ± 20.69* (21)
W/kg	3.24 ± 0.65	2.76 ± 0.59 **	2.98 ± 0.66	2.28 ± 0.48* (21)
W/(kg^0.66^)	10.64 ± 1.87	9.04 ± 1.76**	9.78 ± 1.98	7.64 ± 1.45* (21)
W/(kg^0.51)^	N/A	15.26 ± 2.94	15.26 ± 2.94 (163)	12.60 ± 2.43* (12)
W/(kg^0.35)^	36.29 ± 6.04	N/A	36.29 ± 6.04 (140)	27.77 ± 5.52* (9)
W/cm	0.77 ± 0.13	0.66 ± 0.13**	0.71 ± 0.14	0.59 ± 0.11* (21)
W/BSA	95.11 ± 16.15	80.86 ± 15.50*	87.45 ± 17.31	69.12 ± 12.49* (21)
Test Score	29.71 ± 9.19	32.69 ± 9.49**	31.32 ± 9.45	30.41 ± 8.15 (285)
Below mean	82	73	155	150
Maturity offset	-3.31 ± 0.27	-2.23 ± 0.32**	-2.73 ± 0.62	-2.78 ± 0.59
Age	9.34 ± 0.31	9.38 ± 0.28	9.36 ± 0.29	9.34 ± 0.29 (259)
Sex	N/A	N/A	54% girls	53% girls

BSA = Body surface area.

*p < 0.05: sample v/s excluded.

** *p* < 0.05: boys v/s girls. Below mean: number of participants with a test score below the mean test score. N/A = not applicable.

Table 2 presents the relations between the anthropometric variables, different CRF expressions and academic achievement. Weight expressed allometrically to the sample’s exponent (W/kg^0.35boys/0.51girls^) successfully adjusted the absolute power output (W) for difference in body size, as indicated by its lack of correlation to corresponding anthropometric variables (kg^0.35boys/0.51girls^). All other CRF expressions were significantly correlated with the anthropometric measures. Significant relations between the anthropometric variables and academic achievement were detected in girls only (Table 2), although no interaction was detected between sex and anthropometric variables with academic achievement as the response variable (all p values > 0.05). All CRF expressions had significant relations with each other with no apparent sex difference, ranging from p = 0.598–0.997, with all p values < 0.05 (data not shown).

**Table 2:** Bivariate correlation between key variables.

	Cardiorespiratory fitness expression						AA
Anthropometrics	Max W	W/kg	W/(kg^0.66^)	W/BSA	W/height	W/kg^ƚ^	Score
*Boys (n 140)*							
Age	0.16	0.01	0.06	0.05	0.10	0.12	0.04
BMI	0.16	-0.62*	-0.41*	-0.34*	0.10	-0.12	-0.03
Weight (kg)	0.33*	-0.55*	-0.30*	-0.25*	0.20*	0.03	0.01
Height	0.46*	-0.19*	0.02	0.01	0.26*	0.26*	0.02
BSA	0.34*	-0.54*	-0.28*	-0.24*	0.20*	-0.01	-0.01
Weight^0.66^	0.31*	-0.57*	-0.32*	-0.27*	0.18*	0.01	-0.02
Weight^0.35^	0.32*	-0.56*	-0.31*	-0.26*	0.19*	0.02	-0.01
Maturity	0.38*	-0.12	0.04	0.04	0.22*	0.23*	0.04
*Girls (n 163)*							
Age	0.16*	-0.02	0.04	0.04	0.11	0.07	0.23*
BMI	0.26*	-0.47*	-0.23*	-0.17*	0.21*	-0.12	0.10
Weight	0.42*	-0.42*	-0.15*	-0.10	0.29*	-0.01	0.17*
Height	0.44*	-0.18*	0.03	0.02	0.25*	0.13	0.21*
BSA	0.43*	-0.40*	-0.13	-0.09	0.29*	0.01	0.19*
Weight^0.66^	0.41*	-0.43*	-0.16*	-0.11	0.28*	-0.02	0.18*
Weight^0.51^	0.41*	-0.43*	-0.15*	-0.11	0.28*	-0.02	0.18*
Maturity	0.41*	-0.14	0.04	0.04	0.25*	0.13	0.27*

* p < 0.05.

ƚ = sample’s mass exponents were 0.51 in girls and 0.35 in boys.

AA = Academic Achievement. W = Watts. BSA = Body Surface Area

Table 3 presents the results from all univariate and multivariate linear regression. CRF did not relate significantly to academic achievement, regardless of its expression, in either sex. CRF had the strongest relation when expressed as absolute power output (Max W) in girls (p = 0.058) but weakened when body weight was added to the model (linear regression scaling). The statistical assumption of a zero-intercept was violated in all cases except one, when body weight was expressed allometrically to the calculated sample’s exponent, 0.51 and 0.35 for girls and boys, respectively. Since adding socioeconomic status as a covariate did not impact the association between different CRF expressions and academic achievement and including it would decrease the number of participants, it was excluded from the analyses.

**Table 3:** Regression coefficients between academic achievement and different CRF expressions.

	Boys (n 140)				Girls (n 163)			
CRF expression	B	β	R^2^	B 95% CI	B	β	R^2^	B 95% CI
Max W	-0.03	-0.06	0.01	[-0.11, 0.05]	0.07	0.15	0.02	[-0.003, 0.15]
*Regression scaling*								
Max W	-0.03	-0.07	0.01	[-0.12, 0.06]	0.05	0.09	0.04	[-0.04, 0.13]
logkg	1.06	0.02		[-8.18, 10.29]	7.20	0.19		[-1.71, 16.10]
*Ratio scaling*								
log(W/kg)	-1.76	-0.04	<0.01	[-9.21, 5.68]	0.08	0.02	<0.01	[-6.08, 7.85]
W/cm	-4.80	-0.07	<0.01	[-17.14, 7.53]	8.13	0.11	0.01	[-3.36, 19.62]
*W/BSA*	-0.03	-0.06	<0.01	[-0.13, 0.06]	0.03	0.05	<0.01	[-0.06, 0.13]
*Allometry/ratio*								
W*kg ^-0.66^	-0.28	-0.06	<0.01	[-1.11, 0.54]	0.23	0.05	<0.01	[-0.56, 1.11]
W*kg ^-0.51^					0.25	0.08	<0.01	[-0.25, 0.75]
W*kg ^-0.35^	-0.09	-0.06	<0.01	[-0.35, 0.17]				

* p < 0.05.

W = Watts. BSA = Body surface area. CRF = cardiorespiratory fitness. In linear regression scaling, max Watt and body weight (logkg) were the independent variables in the model.

## Discussion

In this study, we explored the impact of various body-size scaling methods on the relationship between CRF and academic achievement in Icelandic children. In our cross-sectional sample of 4^th^ grade students, we found that academic achievement was not related to cycle-ergometer-measured CRF, regardless of the body-size scaling method. This finding is in line with previous studies that failed to find a relationship between academic achievement and body-size-scaled cycle ergometer measured CRF in 6-8-year-old Finnish children [28] and 9-,12-, and 15-year-old Australian children [29].

Of all the anthropometric variables used in this study, only body weight scaled to the sample’s exponent seems to fully adjust for different body sizes since it did not relate to the corresponding CRF expression. All other variables had a significant correlation to the corresponding CRF expression, therefore favouring either the lighter (by weight) or taller (by height) participants. This could be explained by the higher body fat level amongst the heavier subjects, but it is more difficult to explain the positive association between body height and corresponding CRF expression since CRF is measured on a size-independent cycle ergometer. The violation of the statistical assumption of zero-intercept could possibly serve as an explanation. However, in this study at least, the violation of the statistical assumption does not seem to greatly impact the results since none of the CRF expressions significantly related to academic achievement. There were only some minor differences in the regression coefficients within the ratio-scaled CRF expressions, with a span of 0.04 and 0.09 in boys and girls, respectively. These differences could, however, could play a role in some cases when the results are considered to be either statistically significant or not.

In the current study, body weight correlated significantly with maximal output in the CRF test in both sexes. This is not surprising since a larger body should be able to produce more absolute power due to more lean body mass. The association between body weight and academic achievement has not been explored in previous studies. In addition, increased body weight is also associated with higher academic achievement, but only among girls. The fact that body weight related to both the independent variable (absolute power) and the dependent variable (academic achievement) supports the use of linear regression scaling [26]. This is, to our best knowledge, the first time linear regression scaling is used when these associations are explored. Linear regression scaling involves entering the body size variable as a covariate in the regression model. This impacts the models differently than when ratio scaling is used since it adjusts for the effect body size has on both the independent variable (CRF) and the dependent variable (academic achievement), further clarifying the relationship. However, the result from linear regression scaling did not differ from that from ratio scaling, with no significant association between CRF and academic achievement. However, even though no significant relation was found, there was an interesting difference in the standardized regression coefficients between linear regression scaling and ratio scaling to body weight expression (W/logkg) in girls, i.e., β = 0.09 vs. 0.02. Therefore, in cases where body weight might affect both the independent variable and the dependent variable, linear regression can be considered as a method of choice.

The association between body weight and academic achievement has not been explored in previous studies. The association found in this study among girls is interesting, but a possible explanation is the association body weight has with maturity (in this study, r = 0.52–0.87, p < 0.001). A more mature individual should be more capable to learn, and Kwak et al. [11] did find indications of associations between pubertal status and academic achievement among adolescents. Therefore, the need to adjust for different body sizes may not only apply to CRF but also to academic achievement.

In addition to using the cycle ergometer to estimate CRF, the more commonly used field tests measure time to walk or jog a specific distance, or the number of completed laps before exhaustion [30]. Contrary to the results from the current study, the vast majority of studies using field tests have reported positive findings when exploring the relations between CRF and academic achievement [7,31,32]. This discrepancy was described in a study conducted by Dwyer et al. [29], who compared the results from a cycle ergometer to results in a one-mile run in 9-, 12-, and 15-year-olds. While no significant relations were reported when results from the cycle ergometer were applied to either sex, positive findings were reported when the running-based field test was used to estimate CRF in all age groups of boys and in 12-year-old girls [29]. This indicates that confounding effects of factors associated with body size or maturity only affect the field tests. The more mature individuals should be able to perform better in a field test because increased stride length increases running economy, but increased running economy has been reported without an increase in maximal oxygen consumption [33]. Body size is much less likely to affect performance on a cycle ergometer, as the length of the crank arm is fixed.

CRF is recognized as a key component of physical fitness and has been used as a proxy for physical activity in previous studies. Different CRF levels can be attributed to both genetic and environmental factors, with physical activity being the main influential factor for adults [34,35]. In children, the relation between CRF and physical activity is less clear. According to Armstrong & Baker, both trained and untrained youth can increase their CRF through endurance training, but the relative intensity of the exercise required to show a benefit needs to be higher than that recommended for adults [6]. The relation between habitual physical activity and CRF has been reported to be weak or insignificant [36–38]. CRF may therefore be used as a proxy for high intensity physical activity as it may not accurately reflect habitual physical activity, which may not be intensive enough to induce improvements in CRF in youths.

The strength of the study includes the relatively large sample in comparison to other studies using a laboratory-based CRF test of 4^th^ grade children who completed a valid CRF test and standardized academic tests, administered and reviewed by a third party. This study has some limitations. First, CRF was measured as maximal power output instead of a direct measurement of oxygen consumption. However, power output has a strong correlation with measured oxygen consumption, or r ≥ 0.90, for both sexes [19]. Second, the lack of laboratory measures to estimate body composition prevented the possibility of expressing CRF relative to fat-free mass. Additionally, the cross-sectional nature of the study, rather than an intervention to change CRF and measure the influence it has on the academic achievement of the individual, can be considered a limitation.

## Conclusion

The results indicate that the use of different methods to express CRF, estimated on a cycle ergometer, did not affect its relation to academic achievement for nine-year-old children. Body weight or body size must be considered when the association between CRF and academic achievement is explored. Body weight expressed allometrically to the sample’s exponent when ratio scaling is applied or linear regression scaling may be the method of choice to adjust for different body sizes when exploring the relation between CRF and academic achievement.
